# How to Optimize the Allocation of Anti-epidemic Materials in Public Health Emergencies From the Perspective of Public Economics

**DOI:** 10.3389/fpsyg.2022.851286

**Published:** 2022-04-11

**Authors:** Ziqi Tang, Zhengyi Wang, Yixuan An

**Affiliations:** ^1^School of Government, University of International Business and Economics, Beijing, China; ^2^School of Marxism, Northwest A&F University, Xianyang, China

**Keywords:** anti-epidemic materials, public psychology, COVID-19, public economics, sustainability

## Abstract

During the COVID-19 public health crisis, market failures such as shortage of supplies and soaring prices of anti-epidemic materials – with masks as the core – have occurred. In essence, such anti-epidemic materials have the dual nature of necessities with low elasticity of demand and private products with positive externalities. This research explores the understanding of anti-pandemic materials and how different initiatives, and evaluation to increase availability of necessary resources can be effective in curbing a pandemic. Market regulation results in a non-Pareto optimal allocation of resources and the difficulty of exerting the positive externalities of products. However, in China, the market failure of anti-epidemic materials was quickly resolved, due to the institutional advantages of socialism with Chinese characteristics, the social responsibility drive of domestic enterprises, and cultural genes that focus on equity and concern for the disadvantaged. The optimal allocation of anti-epidemic materials gave access to exerting efficiency and fairness effects, positive external effects, and public effects.

## Introduction

At the beginning of 2020, a sudden outbreak of COVID-19 swept China, with a total of 31 provinces activating the first-level public health emergency response. On January 31, 2020, the World Health Organization (WHO) declared the novel coronavirus outbreak a public health emergency of international concern. This outbreak became worldwide and rapidly destroyed world systems, with profound impacts for the general population. The devastating effect of this biological disaster was due to the virus’s infectivity, which significantly elevated the world’s mortality rate (Chan, 2020). The initially uncontrollable transmission of COVID-19 caused global socio-economic disruptions, prompting an urgent need to curtail its spread (Sohrabi et al., 2020).

Accordingly, the WHO announced their guidelines for ensuring public health protection. The recommendations for COVID-19 containment encouraged the global government to embrace anti-epidemic measures, such as mask-wearing (Bedford et al., 2020). As a result, during the pandemic, surgical masks have been substantially adopted across areas with high COVID-19 susceptibility (WHO, 2020).

China has typically been able to control infections, thus ensuring its citizens’ health and hygiene. However, the virulence of COVID-19 meant Chinese citizens encountered severe hardship due to the difficulty of containing its spread. As the outbreak occurred close to Chinese New Year, COVID-19 challenged the Chinese population during the incubation period, with symptoms yet to be specified. Presently, China, being the center of the outbreak, has managed to control the devastating effect of the pandemic through protective measures (e.g., medical instruments and testing equipment) (Wilder-Smith and Freedman, 2020). The public health emergency in China has impacted socio-economic developments, thus boosting the need for protective health instruments. Given this context, this study shows that when COVID-19 struck China, the need for additional funds, human power, and anti-epidemic materials (e.g., masks) to respond was paramount (Bai et al., 2020).

However, accompanying the pandemic was a shortage of anti-epidemic materials such as masks, which were in short supply and soaring in price. While threatening people’s health and lives, the pandemic has also profoundly affected the normal operation of the economy and society. Under such circumstances, how to optimize the allocation of anti-epidemic materials such as masks and ensure the safety of people’s lives has become an important issue in preventing and controlling the hazards of the pandemic.

At the end of 2019 in China, and in early 2020 elsewhere, masks became a global necessity. During the COVID-19 pandemic, wearing pharmaceutical masks has been made mandatory by governments across the globe (Li et al., 2020). According to one study, two-thirds of China’s population wore a mask every day during the 2020 phase of the pandemic, thus leading to a substantial increase in mask demand to 900 million (Wang M.-W. et al., 2020). To control COVID-19’s high infectivity, anti-epidemic actions had to be strictly implemented by the Chinese government to maintain its residents’ health (Olufadewa et al., 2021). However, masks are not only a type of anti-epidemic material but are also a scarce resource with public attributes. If masks are mandated as a pharmaceutical intervention, the government is required to ensure the availability of this resource to the general public at the minimum cost. During the pandemic, masks were at first hard to find. From the perspective of the market, the imbalance between supply and demand for masks led to the supply of masks failing to meet the demand, causing a rising price that exceeded the purchasing power of the general public. This imbalance was not just a recurrence of market failures. Issues like the trade-off between individual and collective interests and the weighting of market forces against state forces are also worth considering. Despite the shortage of pharmaceutical demand, countries worldwide have emphasized adopting medical masks as a key measure for combating COVID-19’s severity. As a result, high mask availability effectively eradicates the economic burden, thus universally fulfilling the shortage of mask supply.

Accordingly, within the context of COVID-19, academics have launched preliminary studies on the deployment of epidemic prevention materials represented by masks from a variety of perspectives. Some scholars have studied the supply of masks from the perspective of production. From the perspective of government governance, Liu (2020) conducted an economic analysis of the government’s price-limiting policy for masks and evaluated the impact of the policy. In considering the anti-epidemic material reserve mechanism, Yu (2021) used comparative analysis to analyze the characteristics of the reserve mechanism in the United States and Japan, and then analyzed the implementation path for improving the epidemic prevention material reserve mechanism in China. From a humanistic and ethical perspective, Peng (2020) pointed out that, in major disaster situations, prices formed by free market transactions are not the best way to guide resource allocation or cannot be accepted by social ethics, yet other non-market allocation methods also failed to balance fairness and efficiency.

In particular, effective proactive strategies have profoundly impacted the global health economy, with its full scope yet to be studied (Li et al., 2020). Previous studies focused more on using qualitative methods to study the allocation of anti-epidemic materials such as masks, while ignoring the study of the inherent nature of masks and lacking models to demonstrate this. At the same time, China’s fight against epidemics and pandemics has achieved world-renowned results, especially in the allocation of epidemic prevention materials. China’s experience in combating epidemics needs to be summarized and shared with the world. Therefore, based on public economics, this paper uses a supply and demand model to demonstrate the imbalance between supply and demand and the difference between different groups of people due to the market’s spontaneous allocation of resources in a sudden public health crisis. With the help of factual arguments and logical deductions, this paper analyzes China’s valuable experience in achieving the optimal allocation of epidemic prevention materials and its positive effects.

The high efficacy of masks reduces the risk of being infected (Klompas et al., 2020). As a result, during the pandemic, mitigating the spread of coronavirus has relied strongly on the availability of critical care resources such as masks. Therefore, this study demonstrates the effective use of anti-epidemic equipment for limiting the transmission of deadly transmissible diseases. Indeed, this study holds considerable significance concerning anti-epidemic supply and demand. This study fundamentally covers the suboptimal influence of mask distribution, and therefore highlights the need to prioritize the public’s health over economic benefit. Consequently, the government should efficiently allocate anti-epidemic supplies to the general population, thus ensuring positive public effects.

### Research Objective and Gap

The allocation of resources by the market alone cannot solve the problem of social equity and provide the necessary benefits to disadvantaged groups in society. The market significantly suffers from a state of Pareto allocation inefficiency. This study fills a gap in the research and provides a comprehensive understandings regarding the allocation of resources and social equity.

Notable to the initial COVID-19 pandemic scenario, people will not be infected just because they wear masks, but the more people wear masks, the less people will get infected. Thus, if enough people do not comply with mask-wearing, the wearing of masks becomes useless. China’s private entrepreneurs have effectively exercised their entrepreneurial spirit and taken on social responsibility to respond to the COVID-19 crisis. After the outbreak of COVID-19, they coordinated resources and started production rapidly. Despite a sudden rise in the price of raw materials for the industry chain, a major shortage of production equipment, and a large increase in labor costs, they were able to ensure the production of anti-epidemic materials to cope with market imbalances and contribute their strength in the battle against the epidemic.

Additionally, cultural mechanisms function well as social solidarity in a crisis. People in the disparity pattern pay attention to human affection and refer to the “what goes around comes around” credos, thus forming a chain of material mutual assistance between people. This study reflects on the idea that people in this chain will give material help (such as in the form of sharing resources like masks), and such assistance and support can play an effective role in preventing and mitigating risks.

According to the specific situation of the development of the pandemic, the epidemic prevention materials were eventually reasonably allocated in accordance with a primary, phased, and regional approach. Meanwhile, relying on its political trust, the government was able to stabilize people’s psychological expectations, prevent panic buying, and retain the overall social order, which also brings positive externalities to the entire society. By optimally allocating epidemic prevention materials through these means, governments can create a strong public goods effect and achieve the public good in challenging circumstances.

This paper is structured as follows. Subsequent to the introduction, Section “The nature of anti-epidemic materials” highlights the background of the study, thus presenting detailed information on the intended subject. Section “Materials and methods” proposes the relevant methodology for the study analysis, with section “Study analysis and findings” recording the study results. In addition, section “Conclusion” demonstrates the study findings with references to the prior literature. Finally, the paper offers conclusions regarding the study topic by elaborating the research findings and suggesting future directions.

## The Nature of Anti-Epidemic Materials

This paper will explain the nature of anti-epidemic materials by taking the core material of masks as an example. Before the pandemic, face masks were just standard medical products. The nature of masks, however, have undergone a fundamental change and taken on many new characteristics during the public health crisis caused by COVID-19. Masks have become not only a type of medical product dedicated to epidemic prevention but also necessities in daily life. Though masks are private goods, they can perform the positive externality function of public goods as well.

During the pandemic era, face masks have become the most strategic piece of protective equipment for the public. Hence, numerous countries have witnessed an increase in mask demand, initially not matched by limited supply (Feng et al., 2020). However, facing such deficiency raises the need to adopt vital strategies to effectively facilitate the availability of this critical equipment. The increased deployment of masks favorably reduces the total number of infections and deaths. Hence, the study suggests that rationally allocating anti-epidemic resources among the public can expand this benefit on a wide scale (Worby and Chang, 2020), thus improving public health.

Essential goods with low elasticity of demand: COVID-19 is highly infectious, has a long incubation period, and can be easily transmitted through saliva and the air. At the beginning of the pandemic, there was no specific treatment available. The main methods of controlling infectious diseases are to eliminate the sources of infection, to cut off the transmission routes, and to protect susceptible people. Wearing a mask is an effective way for vulnerable people to protect themselves from infectious diseases and safeguard their health. As a result, masks became extremely important for people in the fight against the virus.

As such, the use of surgical masks became common during the pandemic. Countries across the globe adopted surgical masks on an unprecedented scale, thus curtailing the spread of COVID-19 (Greenhalgh et al., 2020). The use of these medical resources, when widely adopted, limit the spread of the coronavirus, making masks a vital component for lowering transmission of the virus (Rab et al., 2020). Tirupathi et al. (2020) study highlights how the pertinent need for face masks elevates the demand for medical instruments (i.e., masks) (Tirupathi et al., 2020). Indeed, optimal distribution of pharmaceutical equipment among large populations minimize the infectivity rate, essentially due to the increase of anti-epidemic resources.

Undoubtedly, the pandemic had severely affected the world’s socio-economic structures. As of 2020, no vaccine was available to respond to the virus. However, proactive actions such as medical instruments (i.e., masks) limited the healthcare disaster, thus intensifying the global competition for pharmaceutical supplies (Khawaja et al., 2021; Nagurney et al., 2021). The worldwide economy’s decline due to the health crises has potentially harmed the entire epidemic production process. The abrupt market changes during the pandemic event spiked demand for masks, thus causing the government to raise the prices for such equipment (Abdullah et al., 2018; Swanson, 2020). In particular, during the COVID-19 pandemic, the delay in local production of anti-epidemic supplies caused the general public to bear severe health consequences. While the research suggests an urgent need for limiting the spread of COVID-19, problems with anti-epidemic resource allocation initially slowed this process (Worby and Chang, 2020).

Targeted production efforts were needed to curb the shortage of anti-epidemic measures such as masks in the face of this global health crises. The accelerating health repercussions of the virus elevated the need for medical equipment to protect the world’s citizens. However, the production of personal equipment such as masks is limited in many major countries (e.g., China, Germany, the United States, France) (Bown, 2022). As such, during the COVID-19 pandemic, the production of anti-epidemic resources (e.g., masks) has been of immense value for the global public. The research indicates that the supply chain factor involving the production of the protective instrument (i.e., face masks) is a vital contributor to fulfilling the growing need for an additional mask supply (Baldwin and Evenett, 2020).

During the pandemic, the nature of masks has shifted from ordinary medical supplies to consumable daily necessities, with demand becoming less elastic. However, unlike other necessities, masks have had such a special role in the public health emergency that the demand for masks is not very sensitive to price changes. Factors affecting the price elasticity of demand include the degree of product substitution, the wide range of commodity uses, the popularity of commodities, and the price. In the pandemic crisis, the reasons why the demand for masks is not highly sensitive to price changes are: firstly, there are no similar alternatives to masks, and it is difficult for consumers to switch from masks to other items, leaving the market with a very small range of substitute products; secondly, the single use of masks is recommended to prevent viral infections; thirdly, the utility of masks is so strongly understood or mandated that everyone needed a mask to protect themselves against the virus; fourthly, the unit price of masks is low and the price elasticity of demand for masks is very low. Therefore, by their very nature, masks are “essential goods with low price elasticity of demand in a public health crisis.”

Theoretically, masks can be classified as a type of purely private product based on their competitive nature of consumption and exclusivity of benefit. However, during the COVID-19 emergency, masks have a positive externality and can bring benefits to society as a whole once they are used regardless of their clear property rights. The incubation period of the original variant of COVID-19 is up to 14 days from the time of contact to the onset of clinical symptoms in humans and the virus is contagious during the incubation period. Further, the high mobility of the population can lead to rapid and large-scale transmission (Sarfraz et al., 2020). If a person wears a mask, they can be protected from the virus on a personal level and can stop the further spread of the virus, which can enhance the whole society’s efficiency in preventing transmission on a social level. Therefore, masks are a “private good with positive externalities in public health crises.” Indeed, research suggests that policymakers should have consistently recommended the use of masks by the global public to fundamentally curtail the severity of the pandemic (Howard et al., 2021).

The attributes of masks are mainly reflected in two aspects. On the one hand, the demand for masks in a public health emergency is strong and a purely market-based allocation of resources will inevitably lead to uneven distribution, with the highest bidder receiving more masks. On the other hand, although masks are a private product, the government should take an active role in guiding the allocation of resources to better exploit the “public attributes” of masks and increase the overall benefits to society at the critical moment of comprehensive pandemic prevention and control. Private products with positive externalities: It can be evident from Table 1 that products can be classified as public products, quasi-public products, club products, and or private products according to whether consumption is competitive and whether the benefits are exclusive.

**TABLE 1:** The classification of public goods.

	Exclusive	Non-Exclusive
Competitive	Private Products	Quasi-Public Products
Non-competitive	Club Products	Public Products

## Materials and Methods

The quantitative methodology investigated the hypothetical situation of the current pandemic and attempts to explain the balance between supply and demand for masks. This study used secondary data, based on public economics, and a supply and demand model, to demonstrate the imbalance between supply and demand and the difference experiences of various groups of people due to the market’s spontaneous allocation of resources in a sudden public health crisis. With the help of factual arguments and logical deductions, this paper analyzes China’s valuable experience in achieving the optimal allocation of epidemic prevention materials and the positive effects of this. The population of China is divided into two categories: (1) the high-income group who, due to pandemic scenario, will not reduce their demand for masks even if there is a small increase in price; and (2) those at the bottom of the social ladder who struggle to afford the exorbitant price of masks. The information for this study was collected from different platforms such as media, research agencies, newspapers, and policy papers to gain a comprehensive and valuable knowledge regarding the issue. The data used spans the period of January 29, 2020, to May 20, 2020, and different departments were involved in producing the data, such as The Ministry of Industry and Information Technology, National Development and Reform Commission, General Administration of Customs, Ministry of Emergency Management and National Food and Strategic Reserves Administration, General Office of the State Council, Ministry of Transport, National Development and Reform Commission, Ministry of Finance and Ministry of Industry and Information Technology, Joint Prevention and Control Mechanism of the State Council.

## Study Analysis and Findings

Price elasticity of demand refers to how changes to price affect the quantity demand of goods. Although the price elasticity of demand for masks has been relatively small in the pandemic period, this varies greatly for certain groups due to differences in their amount of original capital ownership. In the period of pandemic prevention and control, medical masks are no longer ordinary medical products but instead a daily item required to prevent infection. As they are able to place safety at the top of their value list, the high-income group is willing to buy masks no matter how much they cost to a certain price level. During the pandemic, masks are necessities for them, with a price elasticity of zero, but it will be no longer zero if the price is too high. As for the second group, demand for masks is somewhat elastic as they can only allocate a limited amount of funds. Moreover, although everyone needs the same number of masks every day, at the beginning of the pandemic, the population was in a state of information asymmetry, as cases had yet to peak and there was uncertainty about how the pandemic would unfold. As such, there was a supply demand imbalance in the market for masks, and panic grew among high-income groups. In this case, the higher income groups were willing to pay higher prices and increase the demand for masks since they have enough financial strength to buy excess beyond their normal needs. Consequently, the high-income group is willing and able to buy more masks and their demand will be much higher than that of the low-income group.

**FIGURE 1:**
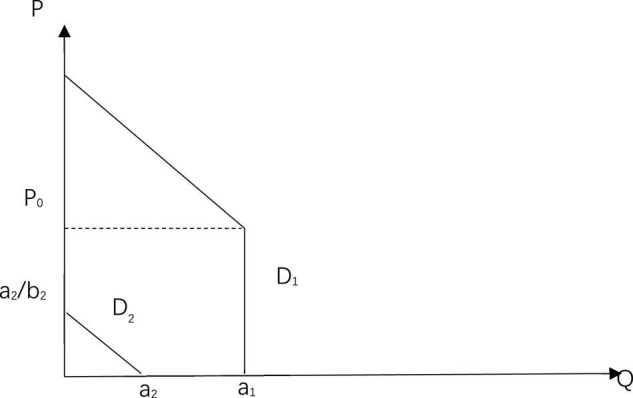
Demand curves for both groups.

**FIGURE 2:**
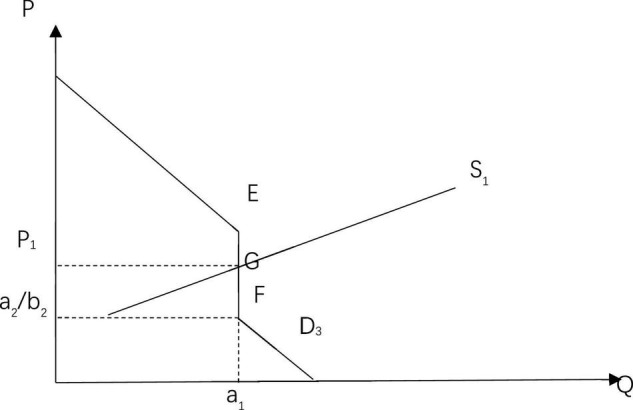
Supply and demand balance for masks under a completely free market configuration.

Thus, it can be assumed that the demand for masks in the first group is represented by the curve: *D*_1_:*Q*_1_ = *a*_1_(*P*≤*P*_0_)*Q*_1_ = *a*_3_−*b*_1_*P*(*P* > *P*_0_); and the demand for masks in the second group is represented by the curve: *D*_2_:*Q*_2_ = *a*_2_−*b*_2_*P*,*a*_1_ > *a*_2_. Both curves are shown in Figure 1. Because it is assumed that there are only these two groups in the whole society, and the aggregate demand curve for private goods is: $Q=\sum_{i=1}^{n}Qi$, the aggregate demand curve for society is: *D*_3_:*Q* = *Q*_1_ + *Q*_2_ = *a*_1_ + *a*_2_−*b*_2_*P*(*P*≤*P*_0_),*D*_3_:*Q* = *Q*_1_ + *Q*_2_ = *a*_3_−*b*_1_*P* + *a*_2_−*b*_2_*P*(*P* > *P*_0_). As shown in Figure 2, there are two inflection points, E and F in D3, at this time. The demand on the abscissa of point E is *a_1*, and the price on the ordinate is *a*_3_−*a*_1_/*b*_2_. The demand on the abscissa of point F is *a_1*, and the price on the ordinate is *a*_2_/*b*_2_. When the price is lower than *a*_2_/*b*_2_, the second group of people are able to buy masks. However, when the price is equal to or higher than *a*_2_/*b*_2_, the total social demand curve is the demand curve of the first group of people, and it only reflects the demand of the first group of people as the second group cannot afford the price of masks currently and their demand for masks cannot be captured in a model with full market assumptions.

At first, in response to the public health emergency, the price of masks were rising everywhere. Therefore, it can be assumed that the price of masks is much higher than the price of *a*_2_/*b*_2_. In the following figures, the aggregate supply curve S1 and the aggregate demand curve D3 intersect at the point G(a_1_,P_1_). At this time, the equilibrium price of masks in the market is P_1_, the equilibrium quantity supplied is a_1_ and *P*_1_*a*_2_/*b*_2_. A balance between supply and demand in the market has been achieved at the expense of the demand for masks for the second group.

## Discussion

### Non-pareto Optimality of Resource Allocation

The market will be regarded as having Pareto inefficiency when there is a significant presence of one social group benefiting while another is suffering. Under spontaneous market regulation, the equilibrium price of masks, P1, is beyond the reach of the low-income group and only the high-income group can afford it, so masks at that price can only satisfy the demand of the high-income group. At this point, the first group in the market benefits and the second group suffers. In addition, the demand for masks in the high-income group exceeds the normal demand of the second low-income group. John Bordley Rawls believes that “Each person possesses an inviolability founded on justice that even the welfare of society cannot override (Sarfraz et al., 2019; Hill, 2021). For this reason, justice denies that it is justified to deprive others of their freedom in order to share the greater interests of some people and it does not allow that the sacrifices imposed on a few are outweighed by the larger sum of advantages enjoyed by many.” In terms of distributive justice, the practices of high-income groups have squeezed out a large proportion of the resources that could otherwise have been purchased by the disadvantaged, resulting in imbalance in the allocation of resources among different groups. As such, the allocation of resources by the market alone cannot solve the problem of social equity and provide the necessary benefits to disadvantaged groups in society. Therefore, the market significantly suffers from a state of Pareto allocation inefficiency (Tao et al., 2021).

### Positive Externalities of the Product Are Difficult to Exploit

With a 14-day incubation period for the first COVID-19 variant, if an undiagnosed infected person wears a mask and goes out, other normal people will not be infected; if ordinary people wear a mask when they go out, even if they meet a suspected patient, they will be protected against the virus due to the protective nature of the mask; if everyone wears a mask outside, the probability of the whole community being infected will drop drastically. Since the allocation of resources is non-Pareto optimal, the second group cannot afford masks due to high prices. The second group that lacks masks may then be at increased risk of being infected by the virus. The more people lack protective equipment such as masks, the wider the spread of the pandemic, and those with masks will also be surrounded by virus carriers (Shehzad et al., 2020; Tuñón-Molina et al., 2021). It is not a given that people will not be infected just because they wear masks, but the more people wear masks, the less people will get infected. A lack of mask-wearing among some groups, coupled with other ways of spreading the virus, will eventually render the wearing of masks useless. The market transaction rule of “success of highest-price-offer” will ultimately increase the likelihood of infection in both groups and increase the rate of infection in the whole society, which will “bring harm to oneself and others” and make “everyone feels insecure,” affecting the overall effectiveness of the fight against the virus (Mohsin et al., 2021; Qu, 2021). Therefore, masks do not effectively exploit the positive externalities of the product by relying solely on market allocation.

### Motivations and Causes for the Optimal Allocation of Epidemic Prevention Materials

When a sudden public health crisis occurs, the demand for epidemic prevention materials, represented in this case by masks, explodes in a short period of time. Under the code of conduct that consumers seek to maximize utility and manufacturers seek to maximize profits, the market, in order to balance the pursuit of the interests of the two, demonstrates its endogenous ability to resolve information asymmetry and rationally allocate resources, and maintain this balance. But this is only a basic state. The reason why this pandemic is called a “magnitude public health emergency” is that the state has gone beyond the usual level of perceived risk. The pandemic has not only changed short-term demand, but also disrupted the original supply chain system (Wang Z. et al., 2020). In other words, the pandemic severely shocked the original order and structure of resource allocation. If we only rely on the market to allocate resources, the Pareto optimal state is difficult to achieve, and the positive externalities of the product are also difficult to exert. As shown in Table 2, China faced the dual dilemma of insufficient masks and low resumption rate in the early stages of the pandemic.

**TABLE 2:** Production of masks at each stage.

Time	Departments	Contents
January 29, 2020	Ministry of Industry and Information Technology	Daily production of masks across the country exceeds 8 million and resumption of work and production reaches 40%.
February 2, 2020	Ministry of Industry and Information Technology	Daily production of masks across the country exceeds 10 million including about 600 thousand N95 masks.
February 3, 2020	Ministry of Industry and Information Technology	The supply and demand of nationwide medical materials reaches a tight balance. As of February 1, the resumed production ratio of urgently needed materials is between 60 and 70%.
February 5, 2020	National Development and Reform Commission	As of February 3, the daily production of masks in 22 key provinces reaches 14.8 million with factory utility up to 67%. China can produce 116 thousand medical N95 masks, 9.98 million other medical masks and 4.71 general masks every day.
February 19, 2020	National Development and Reform Commission	As of February 17, the factory utilization of masks across the country is 109%, approaching 110%.
March 2, 2020	National Development and Reform Commission	1.66 million N95 medical masks are produced per day, and the daily production capability reaches 1.96 million units. This is, respectively, 5.2 times and 12 times the figure of February 1.
April 5, 2020	General Administration of Customs	A total of 3.86 billion masks have been exported from March 1 to April 4.
May 20, 2020	General Administration of Customs	A total of 23.94 billion masks have been exported from April 5 to April 30.

American economist James Buchanan pointed out that when people speak of an existing state as “ineffective” or “flawed”, it implied that there was an ideal, successful state (Burnett and Sergi, 2020). By 2021, China had increased its production capacity of anti-epidemic materials by more than ten times in a very short period of time and began exporting a large number of them overseas. Masks also became available to all income groups in society through a variety of means. The resource allocation of epidemic prevention materials went from a high degree of shortage to a tight balance and finally to a stable balance (Kim et al., 2021). How could the problem of supply and distribution of anti-epidemic materials such as masks be solved in such a short period of time? The answer lies in China’s institutional advantage of concentrating power to achieve great things, corporate social responsibility, and the cultural genes of focusing on equity and concern for the disadvantaged.

### Benefits of Socialism With Chinese Characteristics

In a society, individuals have expectations of the collective because the collective can increase the benefits shared by its members. Conversely, the high cost of cooperation between individuals makes it difficult to take actions spontaneously to achieve collective benefits. As a universal and compulsory public organization, the government naturally has the advantage of acting in concert, which can reduce the cost of cooperation. The rational choice of individuals is to rely on collective actions taken by the government to achieve effective coordination. In the case of a public health response, the government allocates anti-epidemic materials to maximize the efficiency of the entire society (M. Wang, 2021). In all, the reason China has been able to optimize the allocation of epidemic prevention materials in the pandemic and succeeded in the prevention and control battle is that it has the significant advantage over other nations of socialism with Chinese characteristics. There are three main aspects of these institutional advantages.

First, China’s government has a strong and unified leadership structure. The leadership of the Communist Party of China is the greatest advantage of socialism with Chinese characteristics. In the pandemic, the party’s centralized and unified leadership smoothed the flow of government orders and realized the strategic layout of coordinating the whole country. As the old Chinese saying goes, the administration was delegated to local officials and the power was centralized. Facing the COVID-19 outbreak, President Xi Jinping, also the general secretary of the Communist Part of China (CPC) Central Committee and the chairman of the Central Military Commission, personally took command and made preparations. On January 7, 2020, Xi Jinping presided over a meeting of the Standing Committee of the Political Bureau of the CPC Central Committee and issued instructions on the prevention and control of a possible pneumonia epidemic. Central Committee of the CPC and the State Council quickly established a joint prevention and control mechanism and set up a multi-ministerial coordination mechanism platform to macro-control the development of the epidemic through administrative means. They also set up working groups on epidemic prevention and control, medical treatment, and scientific research, with comrades of relevant ministries and commissions as team leaders to clarify responsibilities and divide the work, forming an effective joint force to prevent and control the epidemic. Under the overall plan of the State Council, the Ministry of Industry and Information Technology (MIIT) made every effort to organize enterprises to resume work and production, strengthen the unified dispatch of key materials, and supervise the whole process from production to delivery of key materials; the Ministry of Transport made decisions to block the transmission of the virus while ensuring the continuity of the transport network, the channels for emergency transport, and the transport of goods and materials essential for work and daily life, and prioritized and expedited free road access for emergency transport vehicles, in order to ensure the smooth transportation of anti-epidemic materials. As shown in Tables 3, 4, macro-control during the epidemic period allowed the country to ensure the whole country worked together and to mobilize resources for major undertakings, which provided an institutional guarantee for winning the battle against the epidemic.

**TABLE 3:** Allocation of anti-epidemic materials.

Time	Departments	Files/Contents
January 29, 2020	Ministry of Emergency Management and National Food and Strategic Reserves Administration	Channel 3000 tents, 20000 cotton quilts and 20000 cotton coats that are in urgent need from central reserve system to Hubei province
February 3, 2020	Ministry of Emergency Management and National Food and Strategic Reserves Administration	Channel 3000 tents, 10000 cotton coats and 3000 cots that are in urgent need from central reserve system to Hubei province
February 5, 2020	Ministry of Emergency Management and National Food and Strategic Reserves Administration	Channel 15000 tents, 30000 cotton coats, 30000 cotton quilts and 20000 cots that are in urgent need from central reserve system to Hubei province
February 7, 2020	National Development and Reform Commission	“Decree No. 30 of the National Development and Reform Commission of the People’s Republic of China” takes the approved production capacity of each region as the base, and implements the deployment according to the ratio of medical N95 masks deployment retention ratio of 7:3 and non-N95 masks deployment retention ratio of 3:7.
February 18, 2020	Ministry of Emergency Management and National Food and Strategic Reserves Administration	Channel 50000 cotton quilts and 10000 cots that are in urgent need from central reserve system to Hubei province

**TABLE 4:** Relevant policy documents.

Time	Departments	Files/Contents
January 30, 2020	General Office of the State Council	Urgent notice of the General Office of the State Council on organizing the resumption of work and production and scheduling arrangements for the production enterprises of key epidemic prevention and control materials
January 30, 2020	Ministry of Transport	Notice of the Ministry of Transport on Doing a Good Job in the Priority Guarantee of the Emergency Transportation of Materials and Personnel for the Prevention and Control of the Pneumonia Epidemic Caused by the Novel Coronavirus Infection
February 2, 2020	Ministry of Transport	Urgent Notice of the Ministry of Transport on Effectively Guaranteeing the Smooth Traffic of Emergency Material Transportation Vehicles for Epidemic Prevention and Control
February 10, 2020	National Development and Reform Commission, Ministry of Finance and Ministry of Industry and Information Technology	Notice on Giving Full Play to the Role of the Government’s Reserves and Supporting the Increase in Production and Supply of Short-cut Materials in Response to the Epidemic
February 13, 2020	Joint Prevention and Control Mechanism of the State Council	The State Council’s joint prevention and control mechanism has stepped up policy coordination and material deployment, giving priority to ensuring the needs of key areas.

Second, China’s government has a people-oriented concept of governance. The leadership of the CPC is the defining feature of Chinese socialism. The fundamental purpose of the Communist Party of China to serve the people wholeheartedly makes it always put the people at the center, with their interests and health at the forefront during epidemic prevention (Qian, 2021). The COVID-19 outbreak is sudden and highly contagious, posing a serious threat to health and lives of the people. On January 20, 2020, President Xi gave important instructions on fighting the novel coronavirus. He emphasized that people’s lives and health must come first, and resolute efforts should be taken to stem the spread of the virus.

Third, China’s government has a modern state governance system and capacity. China’s socialist market economy system follows the general laws of market economy operation, fully reflects the effectiveness and modernization of the national governance system and governance capacity, and reflects the decisive role of the market in the allocation of resources. Meanwhile, the government’s macro-control role was effectively brought into play, highlighting the advantages of the socialist system. The socialist market economy combines the cohesive power of socialism with the high efficiency of the market in allocating resources, scientifically using administrative, monetary, and fiscal instruments to effectively ensure the Party’s leading and central role of overseeing the overall situation and coordinating all parties (Chen, 2021). An economic system with Chinese characteristics can overcome the adverse consequences of spontaneous market regulation. Under the overall macro-regulation, the country’s strengths coalesce, and the overall economic situation is able to remain relatively stable over a longer period of time.

### Significance of Corporate Social Responsibility

The state-owned enterprises and private enterprises are the main components of Chinese enterprises. During the pandemic, Chinese state-owned enterprises rushed ahead and played a key role while private enterprises demonstrated their entrepreneurial spirit and assumed risks, with both taking on corresponding social responsibilities.

State-owned enterprises in the public-owned economy are the important material and political foundations for socialism with Chinese characteristics, which bear both economic and social responsibilities. State-owned enterprises play a leading role in the development of the national economy and are an important institutional guarantee for pooling all resources to complete major missions such as fighting COVID-19 (Zhang, 2021). Taking advantage of its dominant position in key areas of the national economy, state-owned enterprises provide a strong supporting force for the stable development of society in crisis periods. State-owned enterprises were at the forefront of the battle against the epidemic and took on heavy responsibility, as befitting their role as the pillar of the nation, fulfilling their original mission with practical actions. For the purpose of increasing the supply of masks in the market, state-owned enterprises worked on research and development, raw materials, production equipment, and transportation to stabilize mas supply on all fronts (Tanjangco et al., 2021). For instance, AVIC Manufacturing Technology Institute took the initiative to undertake the research and development of a medical flat mask machine; Guangzhou Automobile Group Co., Ltd. took advantage of its high-end manufacturing factories to develop mask production equipment in just five days and delivered 41 production lines in one month; Sinopec Group urgently switched production, built 16 melt-blown nonwovens production lines, and allocated resources in collaboration with relevant government departments, all of which were directed to supply mask manufacturers at affordable prices for making masks; and Gree Group advocated that “to fight the epidemic, we build it,” and put up 1 billion to develop high-end medical equipment.

Entrepreneurship is generally regarded by economists as an important factor of production. Theoretically speaking, academics have yet to form a clear and comprehensive definition of entrepreneurship (Al-Qudah et al., 2022), however, there are three major schools of thought on the topic: the German school emphasizes innovation; the neoclassical school focuses on the entrepreneurs’ risk-taking abilities, adventurous spirits, and the ability to cope with market imbalances; and the Austrian school pays attention to the entrepreneurs’ ability to identify market opportunities (Cardella et al., 2021). This paper adopts the neoclassical entrepreneurship theory to explain the impact of entrepreneurship on China’s COVID-19 response.

China’s private entrepreneurs have effectively exercised their entrepreneurial spirit and taken on social responsibility in the fight against the epidemic. After the outbreak of COVID-19, they coordinated resources and started production as soon as possible. At that time, there was a sharp increase in the price of raw materials for the industry chain, a high shortage of production equipment, and a significant rise in labor costs (Wu and Kong, 2021). The entrepreneurs guaranteed the production of anti-epidemic materials to cope with market imbalances and contributed their strength in the battle against the epidemic. For example, Hebei Baota Medical Equipment Co., Ltd. fully switched to the production of masks, assuming its social responsibility during the emergency. Moreover, Tianjin Benao Apparel Co., Ltd. produced masks, protective clothing, and other anti-epidemic materials at full capacity to support the front line of prevention and control.

### Cultural Genes That Focus on Equity and Concern for the Disadvantaged

China’s traditional culture is ancient and has evolved with the development of the times. The key to this lies in cultural genes, which are the fundamental reason why group behavior occurs, like the genetic function of biological genes. Through replication, renewal, and inheritance, cultural genes are passed on from generation to generation and interact with the unique environment of the social system throughout China’s 5,000 years of culture, ultimately forming a cultural genetic chain with Chinese characteristics. According to Fei Xiaotong, Chinese society is a society of disparities, where people are connected mainly by blood and geographical ties. The organic integration of cultural genes and differential order patterns manifests itself in practice as a cultural mechanism centered on face, relationship, and human affection, as well as a daily social unity mechanism. When emergencies and disasters come, the entire society is greatly impacted, resulting in deviations in social operations and social disorder (Scanlon et al., 2021). However, China’s cultural mechanism is so resilient that it allows the public to work together and respond effectively to crises and disasters, such as participating in the prevention and control of epidemics.

Cultural mechanisms function well as social solidarity in a crisis. People in the disparity pattern pay attention to human affection and adhere to the notion that “what goes around comes around,” thus forming a chain of material mutual assistance between people. People in this chain will give material help when others are in need, and likewise, others will give a helping hand when they are facing difficulties. Such help and support can play an effective role in preventing and mitigating risks (Angaw, 2021). People will offer to give some help to others especially in a crisis. Hence, although there was a clear imbalance between different groups in the fully marketed allocation of masks, people would share the masks they had available with acquaintances in their network, driven by the social solidarity function, effectively replacing the market in allocating resources and optimizing the allocation of epidemic prevention materials.

### Consequences of Optimal Allocation of Anti-epidemic Materials

#### Efficiency and Equity Outcomes

The principles of efficiency and equity are two fundamental principles of resource allocation in the field of economics. The optimal allocation of epidemic prevention materials is likely to achieve both efficiency and equity effects. Firstly, the key role of the centralized and unified leadership of the CPC and its coordination of resources across the nation should be highlighted. The government unified the existing stock of epidemic prevention materials in each region and coordinated the supply chain entities to actively start production rapidly, increasing the efficiency of production of anti-epidemic materials (Ryabokon et al., 2021). Further, according to the specific situation of the development of the epidemic, epidemic prevention materials should be reasonably allocated in accordance with a primary, phased, and regional approach.

#### Positive Externalities

Driven by cultural genes, the people spontaneously distribute epidemic prevention supplies among their acquaintances, expanding the range of groups benefited and exerting greater positive externalities (Morelock and Narita, 2021). Meanwhile, relying on its political trust, the government can stabilize people’s psychological expectations, restrain people’s panic buying behavior, and maintain the relative stability of the overall social order, which also brings positive externalities to the entire society.

#### Public Goods Consequence

The optimal allocation of anti-epidemic materials exerts a strong public goods effect. Society’s basic expectations of government are, in order: efficiency, fairness, and public welfare. Behind the supply and demand of anti-epidemic supplies are the social and public interests of an unspecified majority of people (Shu and Wang, 2021). Further, state-owned enterprises are “the ballast” to ensure that overall supply volumes continue to grow and market prices remain manageable, effectively coordinating and balancing the relationship between the public nature of the product and private profitability. As a result, the optimal allocation of epidemic prevention materials has a strong public goods effect and achieves the public good.

## Conclusion

Through its analysis, this paper draws the following conclusions, which have implications for devising future policies. In public health emergencies, the nature of anti-epidemic materials, with masks as the core, has undergone a fundamental change. They became both necessities with low elasticity of demand and private products with positive externalities. Faced with this unique occurrence, the government needs to seek advantages and avoid disadvantages, so the market mechanism is not allowed to regulate itself, which will cause epidemic prevention supplies to become a scarce resource available only to the highest bidder. Instead, it should use “visible hands” to intervene in the market as is necessary and reasonable, ensuring anti-epidemic materials become “public goods” with universal benefits. There are three main reasons why China’s epidemic prevention materials can be optimally allocated: politically, it is mainly due to the institutional advantages of socialism with Chinese characteristics; economically, it is mainly driven by the social responsibility of enterprises; and culturally, it is mainly because of the cultural genes that focus on equity and concern for the disadvantaged. After the anti-epidemic materials are optimally allocated, they will promote efficiency and equity effects, positive externalities, and the public goods effect.

### Study Implications

This study has significant managerial implications. Policy makers and management should be focused on creation and improvement of planning to ensure the balance between supply and demand. This study offers a new insight regarding the management of tourism services in terms of protection and prevention materials. In dealing with public health emergencies, the government is the central hub and the responsible body, and the degree to which its response to the crisis can meet the basic expectations of the public will directly affect the level of public satisfaction with it. To improve social welfare, the Chinese government has integrated epidemic prevention supplies into the public health service, coordinated the production of multiple parties, and restored the entire supply chain as quickly as possible on the supply side, which, no doubt, met the public demands of the Chinese people.

The study is beneficial to the suppliers, demonstrating that anti-epidemic materials should be given to those with the strongest and most urgent need for supplies, such as front-line medical staff and epidemic prevention and control workers, and people in places where the outbreak is the most severe; in the case of COVID-19, Hubei province. The measures implemented by governments have effectively brought into play the fairness effect of the allocation of epidemic prevention materials. The key finding is that enterprises must focus not only on economic performance but also on social responsibility performance in their development. The study provides insights on an entrepreneurial basis, with the huge inversion of supply and demand for epidemic prevention materials formed a perverse interest relationship. In response to the emergency, because of their entrepreneurial spirits, private entrepreneurs resolutely assumed higher operating costs and greater market risks, overcame numerous difficulties to concentrate on the production of anti-epidemic materials, and strove to shoulder their own social responsibilities and historical missions. This study contributes theoretical knowledge and literature related to anti-epidemic materials prominent during the COVID-19 pandemic. This study is also beneficial to researcher examining the many ways to explain the supply and demand of anti-epidemic materials.

### Study Limitations and Future Research

This study is limited to the supply and demand model to demonstrate the imbalance between supply and demand and the difference between different groups of people due to the market’s spontaneous allocation of resources in a sudden public health crisis. As such, future research should be conducted using other methods to analyze the supply and demand of anti-epidemic material. This study focused on logical deductions to analyze China’s valuable experience in achieving the optimal allocation of epidemic prevention materials and its positive effects. Future research can be conducted on logical reasoning based on induction or abduction to evaluate China’s experience in achieving the optimal allocation of epidemic prevention materials. Therefore, this study is limited to the China region; future research can be conducted on other developed and developing countries.

## Data Availability Statement

The raw data supporting the conclusions of this article will be made available by the authors, without undue reservation.

## Author Contributions

All authors listed have made a substantial, direct, and intellectual contribution to the work, and approved it for publication.

## Conflict of Interest

The authors declare that the research was conducted in the absence of any commercial or financial relationships that could be construed as a potential conflict of interest.

## Publisher’s Note

All claims expressed in this article are solely those of the authors and do not necessarily represent those of their affiliated organizations, or those of the publisher, the editors and the reviewers. Any product that may be evaluated in this article, or claim that may be made by its manufacturer, is not guaranteed or endorsed by the publisher.
